# Integration of transcriptomics, metabolomics, and hormone analysis revealed the formation of lesion spots inhibited by GA and CTK was related to cell death and disease resistance in bread wheat (*Triticum aestivum* L.)

**DOI:** 10.1186/s12870-024-05212-3

**Published:** 2024-06-15

**Authors:** Cong Li, Lei Yan, Qian Liu, Rong Tian, Surong Wang, Muhammad Faisal Umer, Muhammad Junaid Jalil, Md Nahibuzzaman Lohani, Yanlin Liu, Huaping Tang, Qiang Xu, Qiantao Jiang, Guoyue Chen, Pengfei Qi, Yunfeng Jiang, Lulu Gou, Qifu Yao, Youliang Zheng, Yuming Wei, Jian Ma

**Affiliations:** 1 State Key Laboratory of Crop Gene Exploration and Utilization in Southwest China, Sichuan Agricultural University, Chengdu, China; 2 Triticeae Research Institute, Sichuan Agricultural University, Chengdu, China; 3 College of Agroforestry Engineering and Planning, Guizhou Key Laboratory of Biodiversity Conservation and Utilization in the Fanjing Mountain Region, Tongren University, Tongren, 554300 China

**Keywords:** Wheat, Transcriptome, Metabolome, Hormone, Disease resistance

## Abstract

**Background:**

Wheat is one of the important grain crops in the world. The formation of lesion spots related to cell death is involved in disease resistance, whereas the regulatory pathway of lesion spot production and resistance mechanism to pathogens in wheat is largely unknown.

**Results:**

In this study, a pair of NILs (NIL-*Lm5*^W^ and NIL-*Lm5*^M^) was constructed from the BC_1_F_4_ population by the wheat lesion mimic mutant MC21 and its wild genotype Chuannong 16. The formation of lesion spots in NIL-*Lm5*^M^ significantly increased its resistance to stripe rust, and NIL-*Lm5*^M^ showed superiour agronomic traits than NIL-*Lm5*^W^ under stripe rust infection.Whereafter, the NILs were subjected to transcriptomic (stage N: no spots; stage S, only a few spots; and stage M, numerous spots), metabolomic (stage N and S), and hormone analysis (stage S), with samples taken from normal plants in the field. Transcriptomic analysis showed that the differentially expressed genes were enriched in plant-pathogen interaction, and defense-related genes were significantly upregulated following the formation of lesion spots. Metabolomic analysis showed that the differentially accumulated metabolites were enriched in energy metabolism, including amino acid metabolism, carbohydrate metabolism, and lipid metabolism. Correlation network diagrams of transcriptomic and metabolomic showed that they were both enriched in energy metabolism. Additionally, the contents of gibberellin A7, cis-Zeatin, and abscisic acid were decreased in leaves upon lesion spot formation, whereas the lesion spots in NIL-*Lm5*^M^ leaves were restrained by spaying GA and cytokinin (CTK, trans-zeatin) in the field.

**Conclusion:**

The formation of lesion spots can result in cell death and enhance strip rust resistance by protein degradation pathway and defense-related genes overexpression in wheat. Besides, the formation of lesion spots was significantly affected by GA and CTK. Altogether, these results may contribute to the understanding of lesion spot formation in wheat and laid a foundation for regulating the resistance mechanism to stripe rust.

**Supplementary Information:**

The online version contains supplementary material available at 10.1186/s12870-024-05212-3.

## Introduction

Bread wheat is one of the most important grain crops in the world. Lesion mimic mutants spontaneously generate programmed cell death (PCD) or necrotic spots on leaves and other tissues without external pathogen infection and environmental stress [1]. The formation of lesion spots is similar to hypersensitivity reactions (HR) induced by plant pathogens, accompanied by up-regulation of defense-related genes, production of reactive oxygen species (ROS), accumulation of polyphenols and callose, and variation of hormone levels [2–6]. Generally, the spontaneous HR caused by lesion spots enhances the broad-spectrum resistance to plant pathogens [7, 8]. Therefore, lesion mimic mutants are an ideal material for studying cell death mechanisms and mining defense-related genes, especially in grain crops [9, 10].

Numerous lesion mimic genes (or lesion spot genes) have been identified and cloned in rice [11], maize [12], and barley [13]. These genes not only influence the formation of lesion spots but enhance resistance to pathogens by regulating different pathways, including splicing factors [14], transcription factors [15, 16], ion channels [17, 18], hormone regulation [19, 20], and protein kinases [21, 22]. In rice, the *SPL5* gene controlling cell death and defense responses through the splicing of RNA precursors is closely related to the SF3b3 splicing factor [14]. *OsNBL3* encoding a mitochondrion-localized pentatricopeptide repeat protein was found to mainly participate in the splicing of gene *nad5* intron 4, resulting in lesion mimic phenotype with enhanced resistance to biotic and abiotic stresses [23]. *SPL29* gene negatively regulates defense responses by regulating jasmonic acid (JA), abscisic acid (ABA), and ROS pathways [19]. In maize, the *ZmMM1* gene as an MYB transcription repressor can cause a lesion mimic phenotype and confer resistance to northern leaf blight, gray leaf spot, and southern corn rust by negatively regulating the transcription of specific target genes *ZmMT3* [24]. In barley, the *NecS1* gene encoding cationic/proton exchange protein controlling cell necrosis and enhancing stem rust resistance is a homologous gene of *HLM1* in Arabidopsis [17]. Additionally, Cysteine protease RD21A regulated by E3 ligase SINAT4 is required for drought-induced immunity in Arabidopsis [25]. Furthermore, it is worth noting that lots of lesions mimic genes that encode protein kinases and play a crucial role in increasing disease resistance to plant pathogens, including *OsPtila* [26], *OsLSD1* [27], *NLS1* [28], and *Mlo* [29].

Moreover, some metabolite or important regulation pathways can also lead to a lesion mimic phenotype and enhance innate immunity. ATP-citrate lyase plays a critical role in the tricarboxylic acid cycle in plants, and it regulates the downstream key gene *OsSL*, which encodes a P450 monooxygenase protein and is related to mediated pathogen defense response [30]. *Nec3* gene encoding cytochrome P450 is aberrant regulation of PCD and leads to cutin layer instability [31]. The *lls1* gene may function to degrade a phenolic mediator of cell death in maize [32]. Furthermore, the *Les22* gene encoding uroporphyrinogen decarboxylase, which is a key enzyme in the biosynthetic pathway of chlorophyll and heme in plants, plays an important role in the disease resistance response of plants to pathogens [33]. *RLIN1*, encoding a putative coproporphyrinogen III oxidase, is involved in lesion initiation in rice [34]. Besides, some important metabolites also play an essential role in cell death and disease resistance. Downstream of the phenylpropanoid pathway, diverse branches of phenylpropanoid metabolism exist, of which the flavonoid pathway is a major branch against pathogenic bacteria and biotic stress [35]. Overaccumulation of phytoalexins induces lesion spots including phenylpropanoids, lignin, and flavonoids suggesting that their biosynthesis was activated in a lesion-dependent manner [36, 37].

However, due to the complexity of the wheat genome, clone and molecular mechanism analysis of lesion mimic genes have progressed slowly in wheat. Only a few lesion mimic genes have been identified and mapped in wheat, for instance, *lm* [38], *lm1* [39], *lm2* [39], *lm3* [40], and *Lm4* [41]. Recently, *Lm5* was fine-mapped to a narrow region on the chromosome arm 2AL [42]. In this study, a pair of near-isogenic lines (NILs) with and without lesion spots formation was used in transcriptomic and metabolomics analysis to identify differentially expressed genes (DEGs), differentially accumulated metabolites (DAMs), and key pathways. Comparative analysis of transcriptome and metabolome, treatment and measurement of hormones, evaluation of disease resistance, and analysis of real-time quantitative polymerase chain reaction (RT-qPCR) also confirmed that the formation of lesion spots can activate the cell death and defense response by protein degradation pathway. These results may contribute to the understanding of lesion spot formation in wheat and laid a foundation for regulating the resistance mechanism to stripe rust.

## Materials and methods

### Construction and phenotypic evaluation of NILs

The homozygous and stable wheat lesion mimic mutant (MC21) was isolated from an EMS-induced Chuannong 16 (CN16) mutant bank [42]. A pair of NILs (NIL-*Lm5*^W^ and NIL-*Lm5*^M^) was constructed from the BC_1_F_4_ population crossed by MC21 and CN16 in the 2020–2021 growing season (Fig. S1). Then, they were planted in the 2021–2022 and 2022–2023 growing seasons in Wenjiang (103° 51ʹ E, 30° 43ʹ N), Sichuan province, China. The plants were individually sowed and harvested. Specifically, each line was planted in 1.5 m single rows with 0.3 m spacing between rows, with a sowing density of 15 seeds per row, i.e., plants within a row were spaced 0.1 m apart [42]. All field trials were irrigated and managed following the local standard practices [42, 43].

### Field evaluation for stripe rust resistance

Four replications of NIL-*Lm5*^W^ and NIL-*Lm5*^M^ were planted in five rows according to the above sowing method, including two uninoculated replications and the other two replications inoculated with stripe rust during the crop seasons of 2021–2022 and 2022–2023. The inoculum was a urediniospore mixture of the predominant *Puccinia striiformis* Westend. f. sp. *tritici* Eriks. (*Pst*) races, obtained from the Gansu Institute (Lanzhou, China) of Plant Protection [42–44]. Relative chlorophyll was also measured using MultispeQ according to the previous methods [42]. Leaf fungal hyphae WGA-PI staining was conducted in Wuhan Service Biotechnology CO., LTD, to identify stripe rust infection.

Agronomic traits of the NILs were evaluated with and without inoculating stripe rust in the field, including plant height (PH), spikelet number per spike (SNS), spike length (SL), flag leaf length (FLL), spike extension length (SEL), flag leaf width (FLW), total number of tillers (TN), thousand-grain weight (TGW), effective tiller (ET), grain length (GL), and grain width (GW). The evaluation methods for these traits were described in previous studies [42].

### RNA-seq and data analysis

At tillering, leaf samples for RNA-seq were taken from three times, based on lesion spots on the leaves, i.e., stage N, when no spots can be observed; stage S, when only a few spots were observed; and stage M, when numerous spots appeared. Leaf tissues of the NIL-*Lm5*^W^ plants were sampled at the same three stages despite the absence of lesion spots. For each stage, three independent biological replicates were made, and all samples were subjected to RNA-seq analysis at Biomarker Technologies (Beijing, China).

The purity and integrity of the RNA were evaluated, subsequently, cDNA libraries constructed using 1 µg RNA per sample were assessed using the Agilent Bioanalyzer 2100 system. Finally, clean reads were obtained from raw reads by removing low-quality reads, adapters, and ploy-N sequences. Meanwhile, Q20, Q30, and GC content of the clean data were further obtained, and the clean reads were mapped to the CS reference genome (IWGSC_RefSeq_v1.1) utilizing the Hisat2 software tools [45, 46]. Based on the mapping results, the reads of a perfect match were further analyzed and annotated. The DEG analysis was carried out using DESeq2 with an adjusted *p*-value < 0.05 [47]. Gene Ontology (GO) enrichment analysis and Kyoto Encyclopedia of Genes and Genomes (KEGG) pathways analysis were performed by the GOseq R packages [48] and KOBAS software, respectively [49]. The other data analysis was processed and performed with a bioinformatic pipeline tool, BMKCloud (www.biocloud.net) online platform.

### Metabolite extraction and LC-MS/MS analysis

Untargeted metabolomics was performed on stage N and stage S samples of NIL-*Lm5*^W^ and NIL-*Lm5*^M^ (each sample with six independent biological replicates) at Biomarker Technologies. The LC/MS system was used for metabolomics analysis [50]. Principal component analysis (PCA) and Spearman correlation analysis were employed to assess the repeatability of the samples within the group and the quantity control samples [51]. Combining the difference multiple, an OPLS-DA model was adopted to identify the differential metabolites with an adjusted *p*-value < 0.05 [52].

### Correlation analysis of transcriptomic and metabolomic data

The DEGs and DAMs were simultaneously identified as their common pathways in the KEGG database and the analysis was also analyzed using the BMKCloud online platform. Furthermore, the correlation network diagrams were constructed using the Cytoscape software (https://cytoscape.org/).

### Hormone treatment and quantification

All hormones were purchased from Beijing Solarbio Science and Technology Co., Ltd. and diluted according to the manufacturer’s instructions. The plants of NIL-*Lm5*^W^ were treated with salicylic acid (SA) and ABA, and those of NIL-*Lm5*^M^ were treated with gibberellic acid (GA) and cytokinin (CTK, trans-zeatin, tZ), all having three concentrations of 1 × 10^− 6^ mol/L, 1 × 10^− 4^ mol/L, and 1 × 10^− 2^ mol/L [53]. Control plants were treated with water. For each hormone, 50 mL of solution was prepared and sprayed evenly on a single row with a hand-held sprayer. Each row had 20 plants grown according to the above sowing method. Hormone application was conducted three times with intervals of about two weeks. Agronomic traits of hormone-treated plants were evaluated, and relative chlorophyll (photosynthesis) was measured for the NILs using MultispeQ [54]. To verify the hormone treatment results, quantification of endogenous hormones was carried out by Metware Biotechnology Co., Ltd, Wuhan, China. Fresh leaf tissues of NIL-*Lm5*^W^ and NIL-*Lm5*^M^ were harvested at stage S. Phytohormone contents were detected by MetWare (http://www.metware.cn/) based on the AB Sciex QTRAP 6500 LC-MS/MS platform.

### RT-qPCR analysis

Fourteen genes were randomly screened to verify the RNA-seq data, including up-regulated genes (3), down-regulated genes (3), genes for sucrose-to-starch metabolism (4), and defense-related genes (4). A total volume of 10 µL reaction buffer including cDNA template (2.5 µL), SYBR Green Premix pro Taq (5 µL, Accurate Biotechnology, Hunan, China), forward primer (0.5 µL, 10 µmol/µL), reverse primer (0.5 µL, 10 µmol/µL) and DNase/RNase-free water (1.5 µL). The RNase L inhibitor-like protein was selected as the reference gene (Table S1), and RT-qPCR analysis was performed on a CFX96TM Real-Time System (Bio-Rad Laboratories, Inc., Hercules, USA), following a program as previously described [42]. To ensure reproducibility, each sample was performed in four biological replicates and three technical replicates, and the relative quantification formula (2^−ΔΔC^_T_) ± standard error of the mean (SEM) was used to further assess quantitative variation.

### Statistical analysis

IBM SPSS Statistics 26 (SPSS Inc., Chicago, IL, USA) was used to perform the Student’s *t*-test (*P* < 0.05), and the software tool of OriginPro, Version 2021 (OriginLab Corporation, Northampton, MA, USA.) was used for further analysis. The open-resource image processing software Image J (NIH, Bethesda, MD; https://imagej.nih.gov/ij/) was used to assess the fluorescence signal intensity.

## Results

### Agronomic traits evaluation

**Fig. 1:**
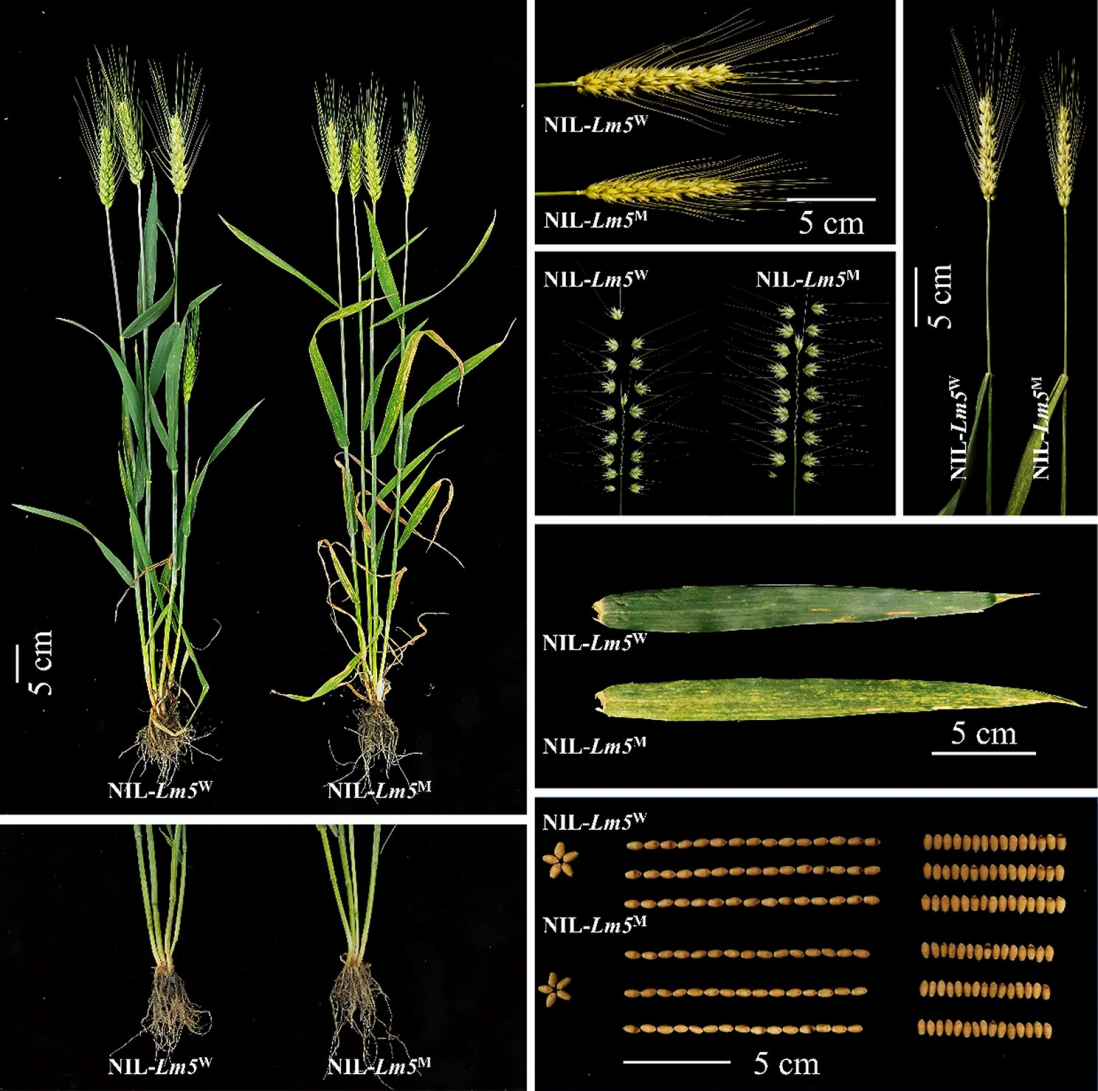
Morphological comparisons between NIL-*Lm5*^W^ and NIL-*Lm5*^M^. Scale bar = 5 cm

There was a great difference in PH, SL, FLL, FLW, SEL, TN, TGW, ET, GL, and GW between MC21 and CN16 (*P* < 0.01), whereas the plants of NIL-*Lm5*^W^ and NIL-*Lm5*^M^ have high similarity in many agronomic traits as is shown in Fig. 1. Compared with the plants of NIL-*Lm5*^W^, those of NIL-*Lm5*^M^ showed significantly decreased PH (5.75%), SL (5.36%), GW (6.93%), and TGW (14.23%), but significantly increased SEL (5.09%) and FLL (30.98%), under field conditions. Furthermore, no significant differences in SNS, FLW, GL, and TN were detected between the plants of NIL-*Lm5*^W^ and NIL-*Lm5*^M^ (Fig. S2a).

### Formation of lesion spots enhanced stripe rust resistance

Under field stripe rust inoculation, *NIL-Lm5*^*M*^ exhibited decreased SL and FLW but increased SEL, FLL, GL, GW, and TGW, when compared with NIL-*Lm5*^W^, whereas no significant differences were detected in PH, SNS, ET, and TN (Fig. S2a). Compared to normal conditions, most of the agronomic traits were reduced ranging from 3.71 to 37.62% with an average of 23.21% in the plants of NIL-*Lm5*^W^ and ranging from 2.15 to 42.84% with an average of 16.82% in the plants of NIL-*Lm5*^M^ under the inoculated condition (Fig. S2a). However, the plants of NIL-*Lm5*^M^ grew normally, whereas those of NIL-*Lm5*^W^ gradually generated cell death under stripe rust inoculation in the field (Fig. S2b). Compared with NIL-*Lm5*^W^, relative chlorophyll in NIL-*Lm5*^M^ decreased by 36.28% under the normal condition but increased by 20.55% under the inoculation condition (Fig. S2c). Compared to normal conditions, relative chlorophyll significantly decreased by 55.63% (NIL-*Lm5*^W^ plants) and 16.05% (NIL-*Lm5*^M^ plants), under the inoculation condition (Fig. S2c).

**Fig. 2:**
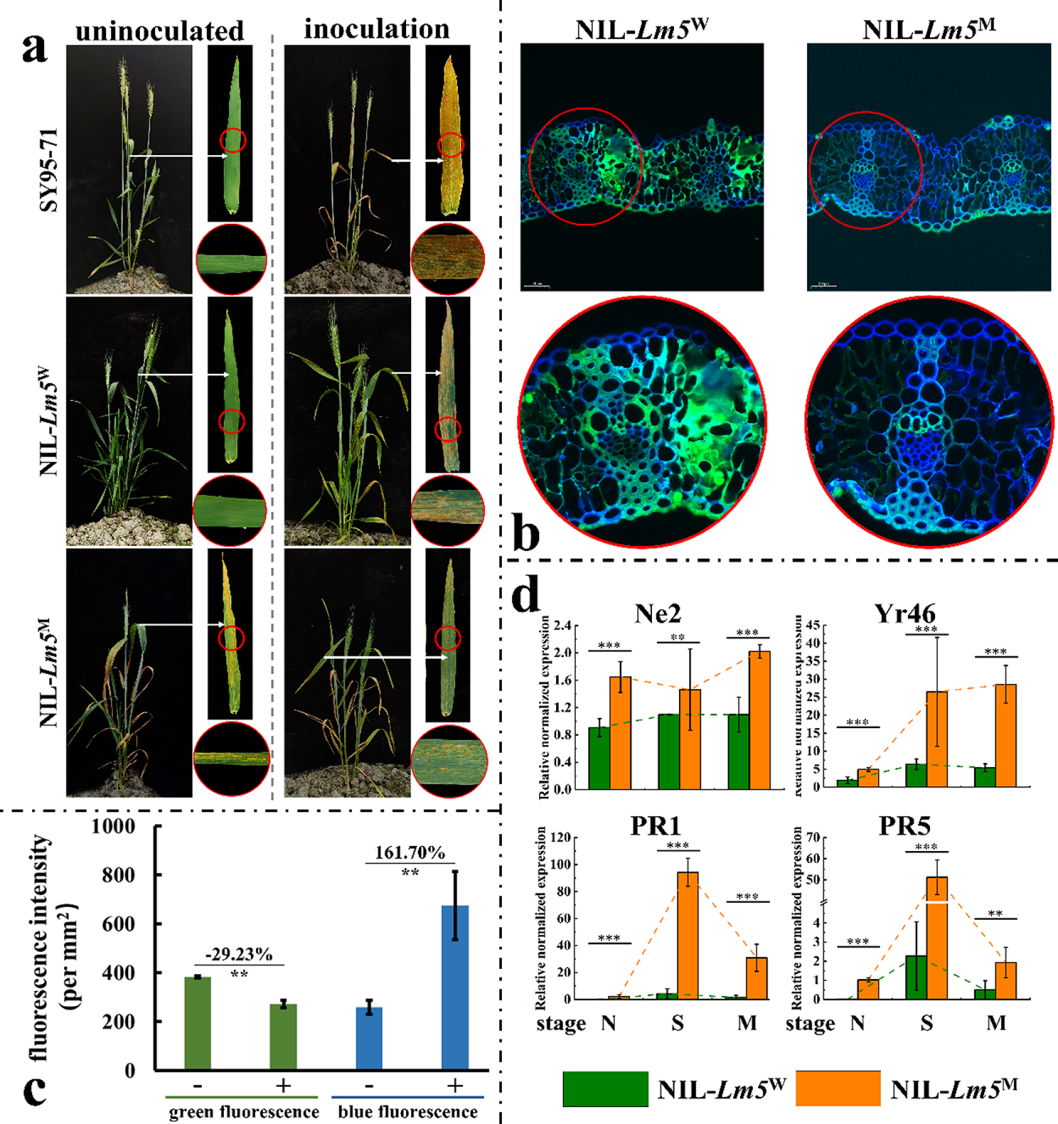
The formation of lesion spots enhanced disease resistance. (**a**) The evaluation of stripe rust resistance between NIL-*Lm5*^W^ and NIL-*Lm5*^M^. SY95-71 is a susceptible control for stripe rust. (**b**) Leaf fungal hyphae WGA-PI staining analysis between the leaves of NIL-*Lm5*^W^ and NIL-*Lm5*^M^. (**c**) The fluorescence signal intensity identified by WGA-PI staining; -: the plants of NIL-*Lm5*^W^; +: the plants of NIL-*Lm5*^M^ (**d**) Expression analysis of defense-related genes at different stages. The error bars represent the SD between biological replicates. Mean fold-changes in the transcript abundance were calculated using the 2^−ΔΔC^_T_ method between biological replicates ± SEMs. N: stage N; S: stage S; M: stage M. **Significant at *P* < 0.01; ***Significant at *P* < 0.001

The plants of NIL-*Lm5*^M^ exhibited significantly enhanced resistance to stripe rust, compared with the plants of NIL-*Lm5*^W^ and SY95-71 (Fig. 2a). Leaf fungal hyphae WGA-PI staining showed that stripe rust hyphae observed in leaves of NIL-*Lm5*^W^ were greater than NIL-*Lm5*^M^ (Fig. 2b). The fluorescence signal also verified that the green fluorescence of the NIL-*Lm5*^M^ decreased by 29.23%, whereas the blue fluorescence increased by 161.70%, compared with the plants of NIL-*Lm5*^W^ (Fig. 2c). Furthermore, RT-qPCR showed that defense response genes (*Ne2*, *Yr46*, *PR1*, and *PR5*) were significantly induced in the plants of NIL-*Lm5*^M^ (Fig. 2d).

### Transcriptome analysis and DEGs identification

To determine the regulatory pathway for the formation of lesion spots, the transcriptome of NIL-*Lm5*^W^ and NIL-*Lm5*^M^ were analyzed in different development stages. A total of 119.76 Gb of clean reads were obtained from the 18 leaf samples. Each sample contained ≥ 5.95 GB of data with Q20 quality scores of ≥ 96.94% and Q30 quality scores of ≥ 92.37% (Table S2). Clean reads ranged from 19,871,883 to 24,162,646, with an average of 22,242,522 (Table S2). Most clean reads were mapped to the reference genome sequence (IWGSC_RefSeq_v1.1), including 91.62% ~ 93.59% mapped reads (of which 86.93% ~ 88.57% unique mapped reads and roughly 4.7% multiple mapped reads, Table S3). Furthermore, the saturability of each RNA-seq sample was closer to 1, suggesting the data is sufficient for further analysis.

In addition, only 488 of 266,753 (~ 0.18%) genes were polymorphic between NIL-*Lm5*^W^ and NIL-*Lm5*^M^, suggesting that the NILs have a similar genetic background (Fig. S3a). There was generally a good correlation among data from the 18 samples (Fig. S4a), yet a lower correlation was shown between stage M samples and the rest samples (Fig. S3b, Fig. S4b). The volcano plot suggested that numerous DEGs were also identified between the NILs at the same stages (Fig. S4c). Specifically, 3611 DEGs (2251 up-regulated and 1360 down-regulated) were identified between NIL-*Lm5*^W^ and NIL-*Lm5*^M^ at stage N, 9384 DEGs (4929 up-regulated and 4455 down-regulated) at stage S, and 15,958 DEGs (7781 up-regulated and 8177 down-regulated) at stage M (Fig. S4d). Additionally, numerous DEGs were identified between different stages of the same plant, e.g., 6691 DEGs (2261 up-regulated and 4430 down-regulated) were identified between stage N and stage S, 975 DEGs (282 up-regulated and 693 down-regulated) between stage S and stage M, and 10,781 DEGs (4112 up-regulated and 6669 down-regulated) between stage N and stage M at of the NIL-*Lm5*^W^ plants; 8443 DEGs (3141 up-regulated and 5302 down-regulated) between stage N and stage S, 4968 DEGs (2781 up-regulated and 2187 down-regulated) between stage S and stage M, and 15,457 DEGs (7296 up-regulated and 8161 down-regulated) between stage N and stage M at NIL-*Lm5*^M^ plants, respectively (Fig. S4d).

**Fig. 3:**
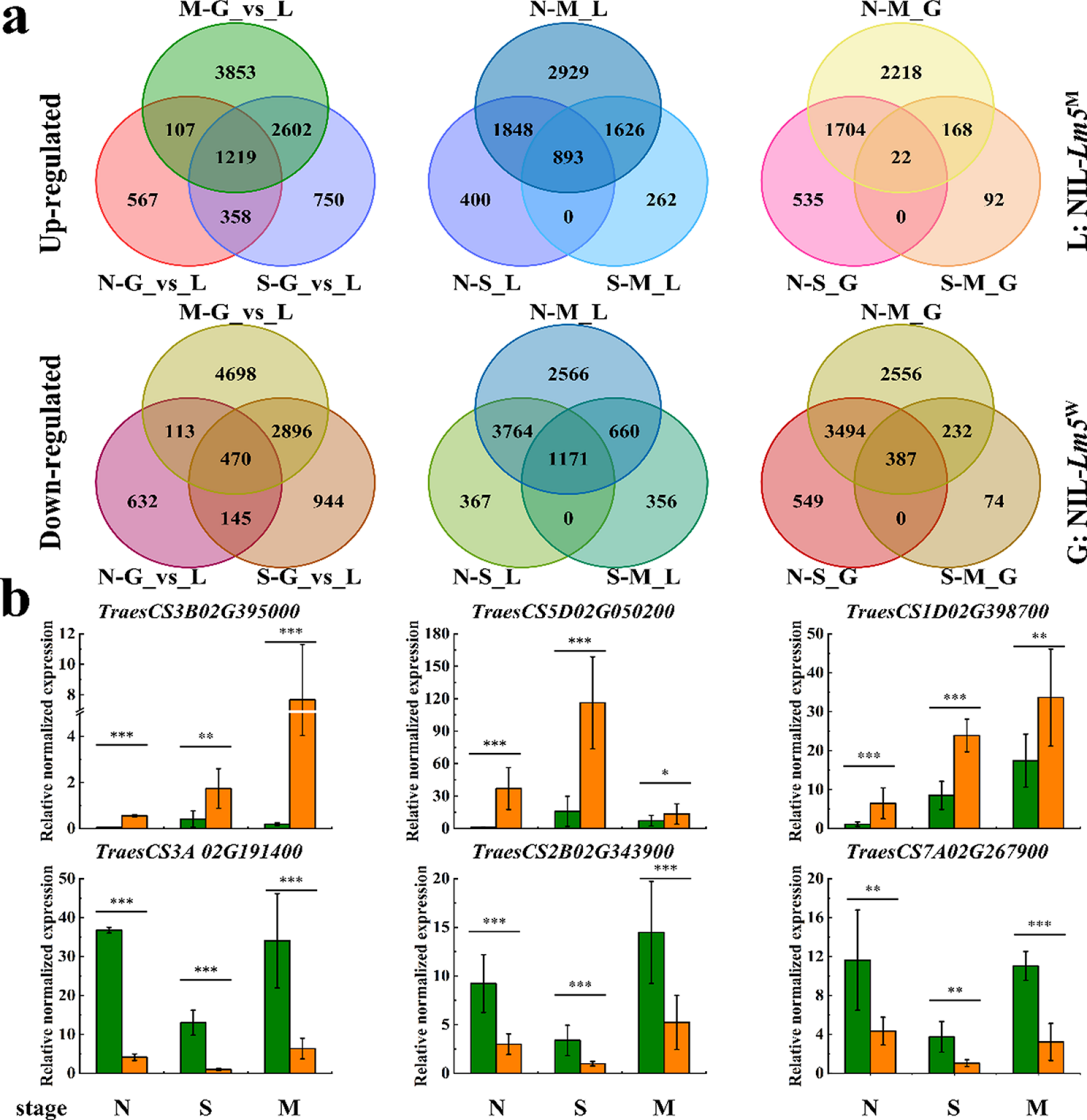
Analysis of differentially expressed genes between NIL-*Lm5*^W^ and NIL-*Lm5*^M^ at different stages. (**a**) Numbers of up-regulated and down-regulated differentially expressed genes at different stages. N: stage N; S: stage S; M: stage M; G: NIL-*Lm5*^W^, L: NIL-*Lm5*^M^. (**b**) Comparison of expression levels of the selected genes using RT-qPCR analysis at different stages. Mean fold-changes in the transcript abundance were calculated using the 2^−ΔΔC^_T_ method between biological replicates ± standard error of the mean. *Significant at *P* < 0.05; **Significant at *P* < 0.01; ***Significant at *P* < 0.001

The up-regulated and down-regulated DEGs were further checked in different comparisons using a Venn diagram, and it turned out that 1219, 893, and 22 of the DEGs were significantly up-regulated, while 470, 1171, and 387 DEGs were down-regulated in these three comparisons, respectively (Fig. 3a). From the DEGs, six genes (three up-regulated and three down-regulated) were randomly selected to verify the RNA-seq data and the results suggested that the transcriptome data are reliable (Fig. 3b). Moreover, GO and KEGG analyses were performed to determine the function of the identified DEGs. Firstly, the top 20 enriched GO terms from all DEGs across different comparisons showed that the carbohydrate metabolic process was most enriched in biological process at stages N and S, whereas translation was most enriched in stage M between NIL-*Lm5*^W^ and NIL-*Lm5*^M^ (Fig. S5a). Chloroplast was most enriched at stages S and M in the cellular component, whereas plastid was most enriched between NIL-*Lm5*^W^ and NIL-*Lm5*^M^ at stage N (Fig. S5b). Importantly, ATP binding was most enriched in all three stages between NIL-*Lm5*^W^ and NIL-*Lm5*^M^ (Fig. S5c). Furthermore, the heatmaps about photosynthesis (Fig. S6a) and carbohydrates implied that a lot of genes were down-regulated to reduce yield and quality (Fig. S6b). RT-qPCR assay confirmed that the expression of genes involving the sucrose-to-starch metabolism was down-regulated at different stages (Fig. S6c).

KEGG enrichment analysis was also performed to analyze the biological functions of DEGs (Table S4). It’s worth noting that plant-pathogen interaction was most enriched in stages S and M, whereas ribosome was most enriched in stage N between NIL-*Lm5*^W^ and NIL-*Lm5*^M^ based on KEGG annotation (Fig. S7, Table S4). Moreover, gene set enrichment analysis (GSEA) also verified that defense-related genes were up-regulation in all three stages between NIL-*Lm5*^W^ and NIL-*Lm5*^M^, including proteasome-mediated ubiquitin-dependent protein catabolic process, proteolysis involved in cellular protein catabolic process, proteasome, defense response to bacterium, flavonoid biosynthesis and ubiquitin-protein transferase activity, peroxisome and proteolysis involved in cellular protein catabolic process (Fig. S8).

Besides, weighted gene correlation network analysis (WGCNA) showed that two important modules (turquoise and blue) were displayed (Fig. S9a). Whereafter, KEGG enrichment analysis was further performed to analyze the biological functions of the genes from the two modules. Specifically, the genes from both modules were most enriched in plant-pathogen interaction based on KEGG annotation analysis (Fig. S9b). In addition, a total of 1831 transcriptional regulators (TR), 6364 transcription factors (TF), and 5467 protein kinases (PK) were identified from the transcriptome analysis results (Fig. S9c). A high proportion of such genes was associated with pathogen resistance, e.g., TRAF and mTERF in TR, AP2/ERF, bHLH, C_2_H_2_, NAC, and MYB in TF, and RLK-pelle_DLSY, RLK-pellw_LRR, and RLK-Pelle_WAK in PK (Fig. S9c).

Altogether, NIL-*Lm5*^W^ and NIL-*Lm5*^M^ have a similar genetic background despite the numerous DEGs (Fig. S3a). The results of KEGG, WGCNA, and GSEA analysis suggested that the DEGs were most enriched in protein catabolic process and defense response to pathogens, implying that the formation of the lesion spots induced cell death and the expression of defense-related genes.

### Metabolome analysis and identification of DAMs

A total of 22,506 peaks were identified using the UHPLC-QTOF/MS system in the 24 samples (each sample has six repeats and two stages), among which 1299 metabolites were annotated (Table S5). Correlation among the samples is high and ranges from 0.65 to 0.99 (Fig. S10a). The PCA presented that the biological repeated data of different stage samples were clustered and separated, implying the experimental processing was effective (Fig. S3c). Moreover, a large number of DAMs were identified between NIL-*Lm5*^W^ and NIL-*Lm5*^M^ in stage N (Fig. S10b) and stage S (Fig. S10c). Specifically, 267 DAMs (two up-regulated and 265 down-regulated) and 505 DAMs (252 up-regulated and 253 down-regulated) were identified between the two NILs at stage N and stage S; and 402 DAMs (341 up-regulated and 61 down-regulated) were identified between stage N and stage S of NIL-*Lm5*^M^ (Fig. S10d).

**Fig. 4:**
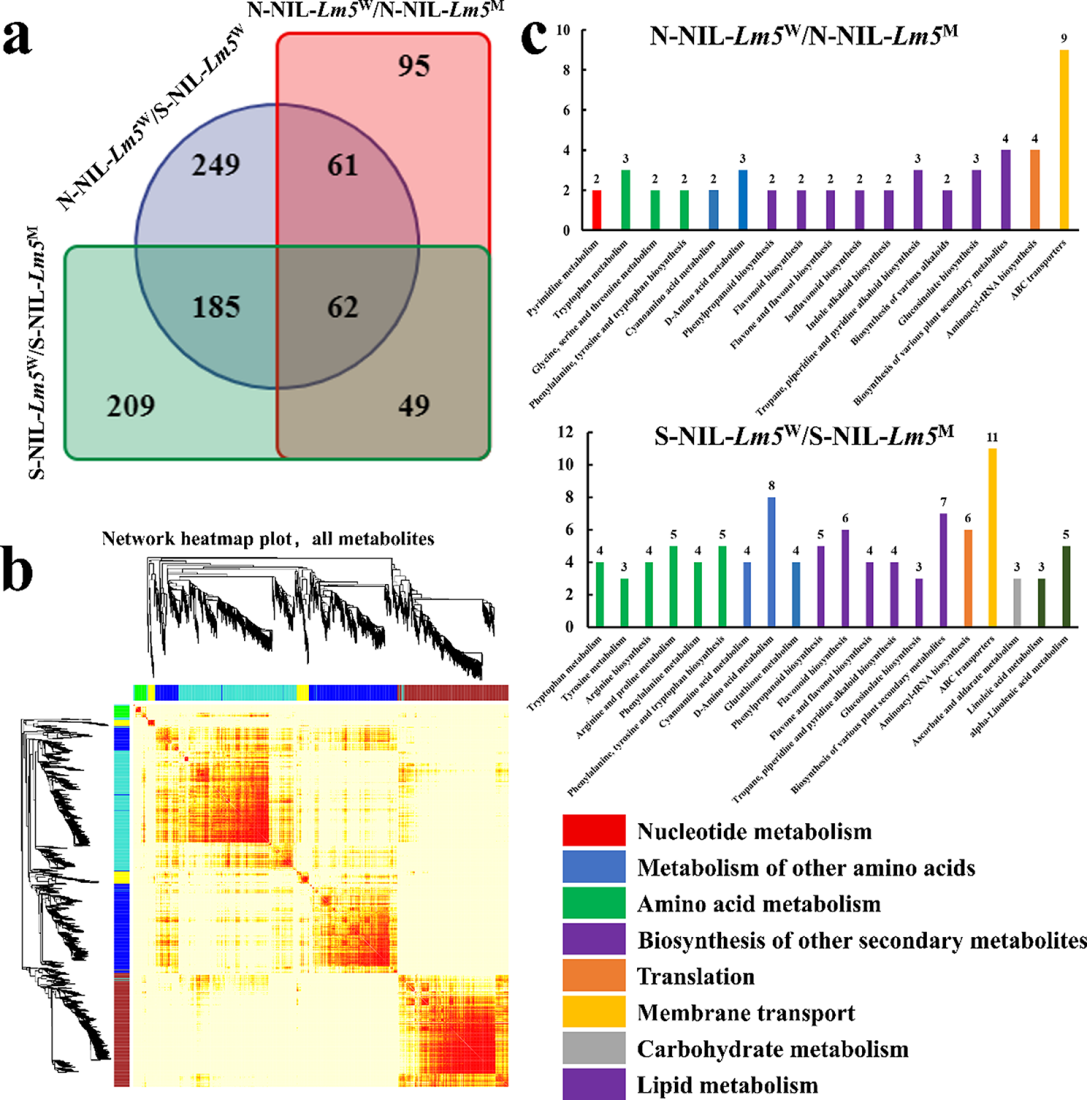
Comparison of differentially accumulated metabolites between NIL-*Lm5*^W^ and NIL-*Lm5*^M^ at different stages. (**a**) Venn diagram of differentially accumulated metabolites in different comparisons. (**b**) The network heatmap of all metabolites. (**c**) KEGG enrichment analysis of differentially accumulated metabolites in different stages

Based on KEGG annotation, 910 DAMs were annotated and were further compared using a Venn diagram. The results suggested that 111 DAMs were identified in the different stages between NIL-*Lm5*^W^ and NIL-*Lm5*^M^, while 62 DAMs were identified between different stages of NIL-*Lm5*^W^ (Fig. 4a, Table S6). WGCNA suggested that five important modules were displayed (Fig. 4b), including green (pos_4812), brown (pos_4355), yellow (pos_9034), turquoise (pos_8713), and blue (pos_8563). KEGG function enrichment analysis showed that the DAMs are most enriched in the biosynthesis of other secondary metabolites, whereas those for membrane transport (ABC transporters) are the highest enriched at the two stages (Fig. 4c). Six pathways were enriched between the two NILs in stage N, including nucleotide metabolism (2 DAMs), metabolism of other amino acids (5), amino acid metabolism (7), biosynthesis of other secondary metabolites (22), translation (4) and membrane transport (9). Seven pathways were enriched between the NILs in stage S, including metabolism of other amino acids (16), amino acid metabolism (25), biosynthesis of other secondary metabolites (29), translation (6), membrane transport (11), carbohydrate metabolism (3) and lipid metabolism (8).

### Comparative analysis of the transcriptome and metabolome

Comparative analysis of the transcriptomic and metabolomic data showed that there were 29 and 60 identical pathways involved in stage N and stage S using KEGG analysis, respectively (Fig. S11a). The most enriched KEGG term from the transcriptomic and metabolomic data was biosynthesis of amino acids, involving 969 DEGs and 19 DAMs in both stage N and stage S (Fig. S11b). Furthermore, glucosinolate biosynthesis, amino sugar and nucleotide sugar metabolism, and valine leucine and isoleucine biosynthesis were also the common enriched pathways at stage N and stage S, which was associated with 969 DEGs and 19 DAMs, 709 DEGs, and 2 DAMs, and 70 DEGs and 3 DAMs, respectively (Fig. S11b). Correlation network diagrams were established to document the relationship among DEGs and DAMs that were commonly enriched in energy metabolism, including ABC transporters and alpha-linolenic acid metabolism pathways both in stage N and stage S (Fig. S11c). Additional, enriched pathways included flavonoid biosynthesis and phenylalanine metabolism at stage N, glycine serine and threonine metabolism, and glyoxylate and dicarboxylate metabolism at stage S (Fig. S11c). Altogether, these results showed that both DEGs and DAMs were enriched in the biosynthesis of amino acids in stage N and stage S, whereas numerous DEGs were enriched in the oxidative phosphorylation pathway, further suggesting that the formation of lesion spots may be associated with cell death.

### Hormone treatment and quantification

**Fig. 5:**
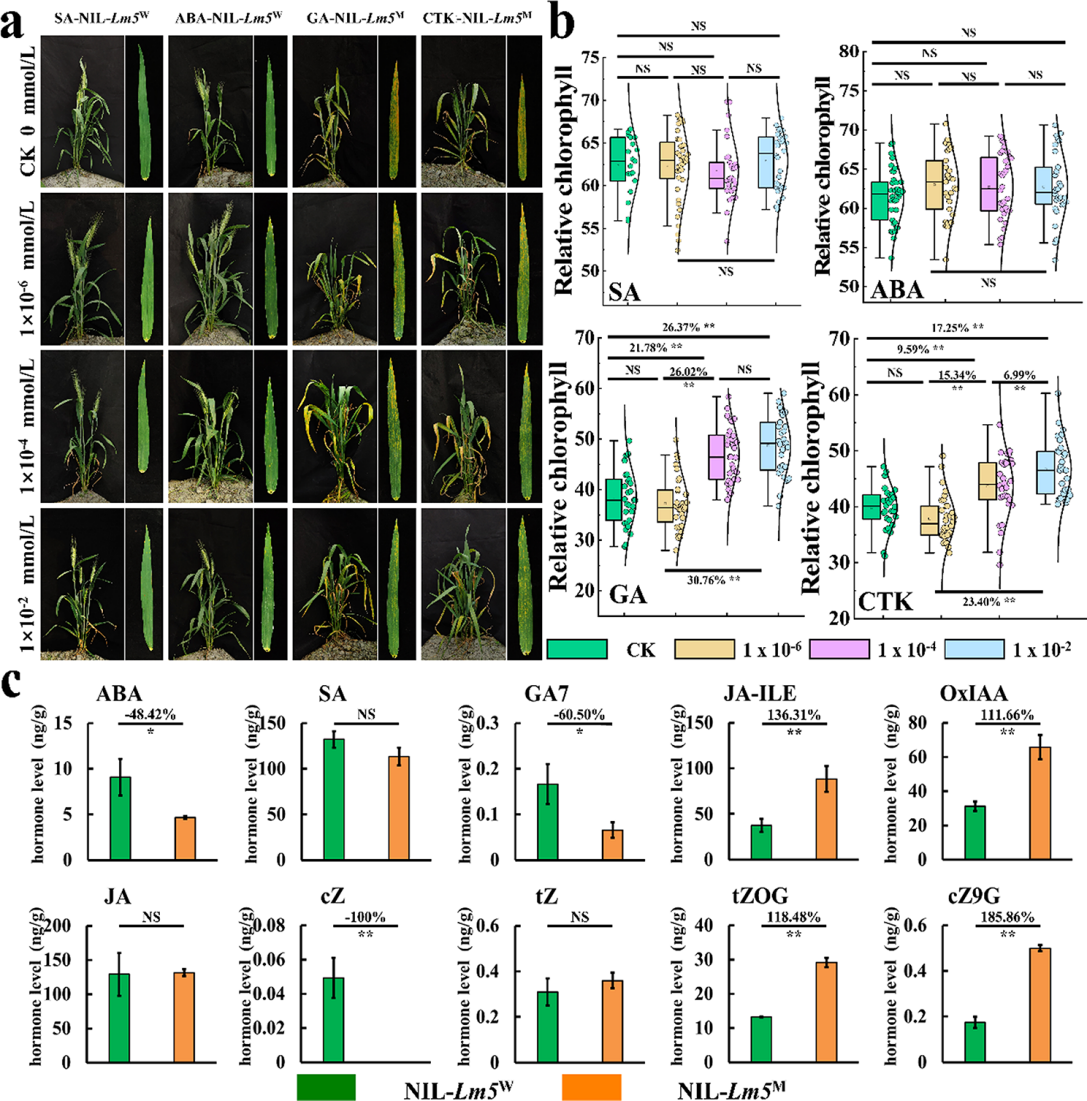
Analysis of different hormone treatments. (**a**) The effect of lesion spot production after treatments with SA, ABA, GA, and CTK. (**b**) The effects of relative chlorophyll between NIL-*Lm5*^W^ and NIL-*Lm5*^M^ after treatments with SA, ABA, GA, and CTK. (**c**) The measurement of different hormones and hormone derivatives between NIL-*Lm5*^W^ and NIL-*Lm5*^M^. JA-ILE: Jasmonoyl-L-isoleucine; OxIAA: 2-oxindole-3-acetic acid; tZOG: trans-Zeatin-O-glucoside; cZ9G: cis-Zeatin-9-glucoside. NS is significant at no difference; *Significant at *P* < 0.05; **Significant at *P* < 0.01

Different hormone treatments exhibited their different effects on the development and growth of the NIL plants (Fig. 5a, Fig. S12a). No differences were detected for relative chlorophyll between the treated vs. untreated NIL-*Lm5*^W^ plants using SA and ABA (Fig. 5b). However, the growth and development of NIL-*Lm5*^M^ can be delayed by spraying exogenous GA and CTK (Fig. 5a, Fig. S12a). Besides, the area of cell death gradually decreased under the GA and CTK treatments, implying that foliar spray of GA and CTK greatly mitigated the formation of lesion spots in NIL-*Lm5*^M^ plants (Fig. 5a, Fig. S12a). Additionally, relative chlorophyll increased significantly after treatments with GA (21.78% ~ 30.76%) and CTK (6.99% ~ 23.40%) at concentrations higher than 1 × 10^− 6^ (Fig. 5b). The evaluation of agronomic traits suggested that SEL, SL, PH, SNS, FLW, and FLL reduced compared to CK after SA and ABA treatments, whereas the opposite trend was observed after GA and CTK treatments (Fig. S12b).

Hormone quantification results showed that the concentration of ABA, cis-Zeatin (cZ), and gibberellin A7 (GA7) decreased by 48.42%, 100%, and 60.50%, respectively, in the *NIL-Lm5*^*M*^ plants (Fig. 5c). However, the concentration of Jasmonoyl-L-isoleucine (JA-ILE), 2-oxindole-3-acetic acid (OxIAA), trans-Zeatin-O-glucoside (tZOG), and cis-Zeatin-9-glucoside (cZ9G) increased by 136.31%, 111.66%, 118.48%, and 185.86%, respectively (Fig. 5c). Compared with NIL-*Lm5*^W^, the genes associated with ABA and CTK were down-regulated in NIL-*Lm5*^M^ (Fig. S12c).

## Discussion

### The formation of lesion spots inhibits the infection of stripe rust

The lesion spot formation could enhance resistance to strip rust and lead to cell death [42]. The formation of lesion spots induced numerous resistant genes (R gene) and GSEA results also verified that many proteins were degradative by the ubiquitination pathway. For instance, the JAZ protein family as key regulators of jasmonate signaling and JA-ILE conjugate promotes physical interaction between COlI and JAZ proteins [55, 56]. Jasmonate ligands promote the binding of the SCF^COlI^ ubiquitin ligase and subsequent degradation of the JAZ repressor protein resulting in PCD [55, 57]. Furthermore, leaf fungal hyphae WGA-PI staining suggested that the stripe rust pathogen was suppressed when the lesion spots formed in NIL-*Lm5*^M^, which was in agreement with previous findings that the formation of lesion spots can prevent the infection of pathogenic bacteria [42]. Moreover, the expression of defense-related genes is the most important regulation of plant basal immunity (HR) and cell death.

In the present study, a combined analysis of the transcriptome and metabolome suggested that DEGs and DAMs are enriched in the carbohydrate metabolic process and plant-pathogen interaction, suggesting the formation of lesion spots is related to other pathways, including energy metabolism [30, 34], flavonoid [35], and transcription factors [58]. For example, mitochondria and chloroplast as important organelles not only provide energy for plants’ growth and development but also are closely related to disease resistance. Previously we have found that a large amount of chloroplast structure has degenerated in leaves, and mitochondria are barely detectable in the mutant plants [42]. Therefore, mitochondria and chloroplasts may be involved in the cell death and resistance response. Furthermore, it has been reported that the genes from mitochondria and chloroplasts are related to disease resistance in rice and wheat [30, 59]. For example, *RLIN1*, encoding a putative coproporphyrinogen III oxidase in the tetrapyrrole biosynthesis pathway, is involved in the formation of lesion spots in rice [34]. *OsACL-A2* negatively regulates innate immune responses by reducing ATP-citrate lyases enzymatic activity and accumulates high ROS in rice [30]. *TaISP* protein as an effector protein from wheat stripe rust fungus targeting chloroplasts suppresses chloroplast function and plant basal immunity by reducing callose deposition and the expression of defense-related genes [59].

**Fig. 6:**
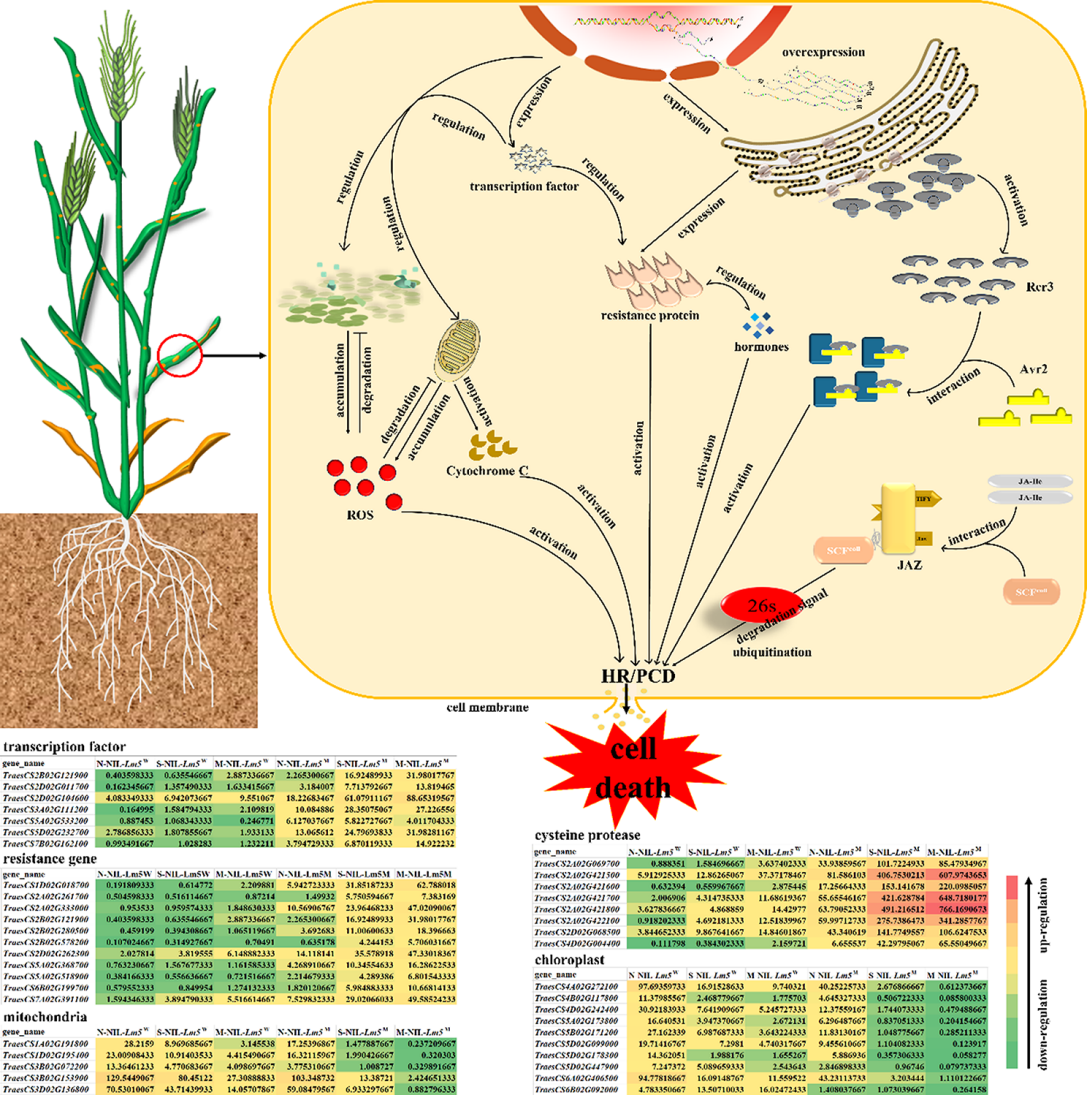
A proposed and simplified pathway for the formation of lesion spots and the subsequent cell death

Besides, important metabolite and transcription factors also regulate cell death and resistance to pathogens. For example, Phenylpropane metabolism includes a lignin synthesis pathway, flavonoid synthesis pathway, and procyanidin-specific synthesis pathway. Especially, lignin and flavonoids are specialized metabolites frequently reported as involved in plant defense against biotic stresses, thus, their biosynthetic accumulation in the formation of lesion spots may enhance the resistance to stripe rust [35]. Furthermore, transcription factors play an important role in modulating the transcriptional and resistance response of the cell [15, 60]. Some transcription factors not only regulate the expression of defense-related genes indirectly but are involved in resistance to pathogens directly. For example, ZmMM1 acts as an MYB transcription repressor and negatively regulates the transcription of target genes, conferring resistance to northern leaf blight, gray leaf spot, and southern corn rust in maize [24]. MACD1, which is an AP2/ERF transcription factor, participates in phytotoxin-triggered cell death and acts downstream of ethylene signaling, suggesting that MACD1 positively regulates factors affecting cell death and resistance [16]. ​Additionally, caspase activation plays a central role in the execution of apoptosis, and many genes coding cysteine protease were overexpressed in the current study. Therefore, based on these results and previous studies a simple related pathway for leading to cell death (lesion spots) and inducing HR is proposed (Fig. 6). The application of these materials/genes has a positive significance for enhancing disease resistance for breeding in the future.

### Amino acid metabolism disorder resulted in agronomic trait difference

A lot of DEGs and DAMs are enriched in amino acid metabolism. Energy metabolism metabolites play a role in the development and growth of wheat, especially in maintaining vital activities [61]. For example, due to the degradation of chloroplasts, the decrease in photosynthetic capacity, and the lack of photosynthetic products led to the insufficient synthesis of a large number of important substances, which affects the expression of genes and the production of metabolites, such as glucose and fructose. Furthermore, the TCA cycle is not only a common pathway for oxidative decomposition of sugars, fats, and amino acids, but an important biosynthetic hub for their mutual transformation in mitochondria [61].

Besides, despite our continuous backcross the constant purification of genetic background and only 0.18% of genes were polymorphic between NIL-*Lm5*^W^ and NIL-*Lm5*^M^, many DEGs and DAMs were still identified between NIL-*Lm5*^W^ and NIL-*Lm5*^M^ in the same stages, because of changes in photosynthesis and carbohydrates occurred by cell death. On the other hand, auxin was oxidized to acetic acid, which leads to the inactivation of auxin, thus affecting the growth and development of plants. That may be why significant differences were detected in some agronomic traits such as PH, SL, and TGW during agronomic trait evaluation between NIL-*Lm5*^W^ and NIL-*Lm5*^M^.

### GA and CTK inhibited the formation of lesion spots and had a positive effect on agronomic traits

Hormones can regulate plant growth and development, such as GA and CTK can promote plant growth, whereas, ABA and JA can promote plant premature senility and enhance resistance. The formation of lesion spots is inhibited by spraying GA and CTK for lesion mimic mutant plants in the field, whereas it is unaffected by spraying JA and ABA for the normal plants. One important reason may be the decrease of endogenous hormone content, such as ABA, GA7, and cZ in the leaves of NIL-*Lm5*^M^ plants. Whereafter, the transcriptome has verified that the genes controlling CTK and ABA were altered. It is worth noting that the content of GA, ABA, and CTK were reduced.

Despite ABA and GA having different effects in plants, mevalonic acid, a terpenoid compound, is their common precursor [62]. Mevalonic acid, which forms GA under long-day conditions and ABA under short-day conditions, is synthesized from acetyl-CoA [63]. Therefore, we suspected the reduction of ABA is caused by the decrease in the content of precursor substances. Previous studies reported that glucosinolate biosynthesis not only plays a major role in regulating plant hormone ABA and the secondary metabolite but is also required as a component in plant defense response against microbial pathogens [64, 65]. Therefore, the decline of the ABA level as an important signal factor may regulate a complicated network of synergistic and antagonistic interactions.

The content of CTK was decreased due to the degradation of mitochondria and chloroplasts. Because isopentenyl pyrophosphate is one of the key substances in the synthesis of CTK. For example, acetyl-CoA is converted to mevalonate and then further converted to isopentenyl pyrophosphate through the mevalonate pathway [66]. Acetyl-CoA is an important substance for the TCA cycle in mitochondria. Additionally, through a series of reactions, pyridoxal pyrophosphate, and glyceraldehyde-3-phosphate can also generate isopentenyl pyrophosphate and dimethylallyl pyrophosphate through the MEP/DOXP pathway in chloroplasts [67].

Hormones are well known as vital regulators to affect growth and development in plants. Generally, some agronomic traits are increased by spraying GA and CTK but decreased by spraying ABA and SA [20, 68], which also agrees well with the current study. The formation of lesion spots was ineffective by spraying ABA and SA in the NIL-*Lm5*^W^ plants, but the level of ABA in NIL-*Lm5*^M^ decreased compared with NIL-*Lm5*^W^.

### Electronic supplementary material

Below is the link to the electronic supplementary material.

Supplementary Material 1

Supplementary Material 2

Supplementary Material 3

Supplementary Material 4

Supplementary Material 5

Supplementary Material 6

Supplementary Material 7

## Data Availability

All data supporting the findings of this study are available within the paper and within its supplementary materials published online. The original RNA-seq data of NIL-Lm5W (normal) and NIL-Lm5M (lesion spots) in wheat leaves are available at the NGDC’s Genome Sequence Archive (GSA) database under BioProject CRA015499.
