# Supplementary material for: Integration of transcriptomics, metabolomics, and hormone analysis revealed the formation of lesion spots inhibited by GA and CTK was related to cell death and disease resistance in bread wheat (Triticum aestivum L.)
Source: BMC Plant Biol. 2024 Jun 15;24:558. doi: 10.1186/s12870-024-05212-3 (PMC11179392; doi:10.1186/s12870-024-05212-3)
Supplement: Supplementary file 7 — Supplementary Material 7 [file 12870_2024_5212_MOESM7_ESM.docx]

**Fig. S1** Schematic diagram of constructing near-isogenic lines.

**Fig. S2** Phenotypic characteristics of NIL-*Lm5*^W^ and NIL-*Lm5*^M^. **(a)** The performance of agronomic traits in NIL-*Lm5*^W^ and NIL-*Lm5*^M^ with and without inoculation; plant height (PH), spike length (SL), spike extension length (SEL), grain length (GL), flag leaf length (FLL), flag leaf width (FLW), spikelet number per spike (SNS), grain width (GW), total tiller number (TN) effective tiller (ET) and thousand-grain weight (TGW). **(b)** The plants of NIL-*Lm5*^W^ and NIL-*Lm5*^M^ with and without inoculation of stripe rust. **(c)** The effects of relative chlorophyll between NIL-*Lm5*^W^ and NIL-*Lm5*^M^ with and without inoculation. NS stands for non-significant, *Significant at *P* < 0.05, **Significant at *P* < 0.01.

**Fig. S3** The evaluation of transcriptomic and metabolomic. (a) The genetic background analysis between NIL-*Lm5*^W^ and NIL-*Lm5*^M^. **(b)** The PCA analysis of transcriptomic. (c) The PCA analysis of metabolomic.

**Fig. S4** Transcriptome analysis at different stages. **(a)** Correlation analysis of samples from different stages. **(b)** Heatmap analysis of gene expression from 18 samples in different stages. **(c)** Volcanic map analysis of differentially expressed genes in different stages. **(d)** The number of differentially expressed genes between different groups; N: stage N; S: stage S; M: stage M; G; the plants of NIL-*Lm5*^W^; L: the plants of NIL-*Lm5*^M^.

**Fig. S5** GO enrichment analysis of differentially expressed genes between different groups. Differentially expressed genes enrichment to biological process **(a)**, cellular component **(b)**, and molecular function **(c)**.

**Fig. S6** The differentially expressed genes analysis related to energy metabolism. The differentially expressed genes analysis related to photosynthetic **(a)** and carbohydrate **(b)**. **(c)** Expression analysis of energy metabolism genes at different stages. Mean fold-changes in the transcript abundance were calculated using the 2^−ΔΔC^_T_ method between biological replicates ± SEMs. N: stage N; S: stage S; M: stage M. NS is significant at no difference, *Significant at *P* < 0.05, **Significant at *P* < 0.01.

**Fig. S7** KEGG enrichment analysis of differentially expressed genes between different groups.

**Fig. S8** Gene set enrichment analysis containing cell death and disease resistance in different comparisons. N: stage N; S: stage S; M: stage M.

**Fig. S9** WGCNA and transcription factor prediction. **(a)** Cluster dendrogram analysis of the transcriptome. **(b)** KEGG analysis of two important groups from the cluster dendrogram. **(c)** transcriptional regulator (TR), transcription factors (TF), and protein kinases (PK) were identified from the transcriptome analysis.

**Fig. S10** Metabolome analysis at different stages. **(a)** Correlation analysis of samples from different stages. Heatmap analysis of differentially accumulated metabolites in stage N **(b)** and stage S **(c)**. **(d)** The numbers of differentially accumulated metabolites were identified in different stages; G; the plants of NIL-*Lm5*^W^; L: the plants of NIL-*Lm5*^M^.

**Fig. S11** Correlation analysis of differentially expressed genes and differentially accumulated metabolites of leaves between NIL-*Lm5*^W^ and NIL-*Lm5*^M^ in different stages. **(a)** The numbers of differentially expressed genes and differentially accumulated metabolites that were enriched in the same pathway at stage N (left) and stage S (right). **(b)** KEGG analysis of differentially expressed genes and differentially accumulated metabolites that were enriched in the same pathway at stage N (up) and stage S (down). **(c)** Correlation network diagram of differentially expressed genes and differentially accumulated metabolites in stage N and stage S.

**Fig. S12** Hormone analysis between NIL-*Lm5*^W^ and NIL-*Lm5*^M^ at the same stage. **(a)** The plants of NIL-*Lm5*^W^ were treated by spraying ABA and SA and the plants of NIL-*Lm5*^M^ were treated by spraying GA and CTK. **(b)** The trend analysis of differentially expressed genes related to hormones from RNA-seq. **(c)** The performance of agronomic traits between NIL-*Lm5*^W^ and NIL-*Lm5*^M^ after treatment with SA, ABA, GA, and CTK, respectively.
